# Influence of Fiber Bragg Grating Spectrum Degradation on the Performance of Sensor Interrogation Algorithms

**DOI:** 10.3390/s141224258

**Published:** 2014-12-16

**Authors:** Alfredo Lamberti, Steve Vanlanduit, Ben De Pauw, Francis Berghmans

**Affiliations:** 1 Department of Mechanical Engineering, Vrije Universiteit Brussel (VUB), Pleinlaan 2, 1050 Elsene, Belgium; E-Mails: Steve.Vanlanduit@vub.ac.be (S.V.); bdepauw@vub.ac.be (B.D.P.); 2 Faculty of Applied Engineering, University of Antwerp, Campus Hoboken Salesianenlaan 90, 2660 Antwerp, Belgium; E-Mail: steve.vanlanduit@uantwerpen.be (S.V.); 3 Department of Applied Physics and Photonics, Vrije Universiteit Brussel (VUB), Pleinlaan 2, 1050 Elsene, Belgium; E-Mail: fberghma@b-phot.org

**Keywords:** fiber Bragg grating, optical sensing, peak detection algorithms, spectral distortion, dynamic measurements, precision and accuracy

## Abstract

The working principle of fiber Bragg grating (FBG) sensors is mostly based on the tracking of the Bragg wavelength shift. To accomplish this task, different algorithms have been proposed, from conventional maximum and centroid detection algorithms to more recently-developed correlation-based techniques. Several studies regarding the performance of these algorithms have been conducted, but they did not take into account spectral distortions, which appear in many practical applications. This paper addresses this issue and analyzes the performance of four different wavelength tracking algorithms (maximum detection, centroid detection, cross-correlation and fast phase-correlation) when applied to distorted FBG spectra used for measuring dynamic loads. Both simulations and experiments are used for the analyses. The dynamic behavior of distorted FBG spectra is simulated using the transfer-matrix approach, and the amount of distortion of the spectra is quantified using dedicated distortion indices. The algorithms are compared in terms of achievable precision and accuracy. To corroborate the simulation results, experiments were conducted using three FBG sensors glued on a steel plate and subjected to a combination of transverse force and vibration loads. The analysis of the results showed that the fast phase-correlation algorithm guarantees the best combination of versatility, precision and accuracy.

## 1. Introduction

Fiber Bragg grating (FBG) sensors can be dated back to 1978, when Hill *et al*. [1] discovered that refractive index variation (*i.e.*, gratings) can be formed in optical fibers. Since then, the interest in the fabrication and application of FBG sensors has exponentially grown [2,3]. Today, FBG sensors are adopted for probing a variety of measurands, such as strain, temperature, pressure, erosion and even magnetic fields [3,4]. Compared to conventional electrical sensors, FBG sensors offer a number of attractive advantages. First, they are immune to electromagnetic interference. Second, they can be integrated within materials in a minimally-intrusive manner thanks to their small size and weight. Furthermore, chains of serially-connected FBGs can be straightforwardly multiplexed to enable quasi-distributed measurements. These advantages led to the introduction of FBG sensors in many applications [5,6]. FBG sensors use the so-called Bragg condition, according to which the Bragg wavelength λ*_B_* of the light reflected by the grating obeys the following law:

(1) $$\lambda_{B}=2n_{\text{eff}}\Lambda$$

where *n*_eff_ is the effective refractive index of the fiber and Λ is the grating period. The wavelength of the reflected light (the Bragg wavelength) is sensitive to the magnitude of the measurand at the sensor location. Therefore, it is possible to retrieve information about the measurand by tracking the Bragg wavelength shifts. To accomplish this task, many interrogation schemes have been developed in the last few years [7,8]. At the same time, several peak detection algorithms have been proposed in the literature, from conventional techniques, such as the maximum detection (MD) and centroid detection (CD) algorithms, to more recent and advanced neural network [9] methods and correlation-based techniques, such as the auto- and cross-correlation algorithms (AC, CC) [10,11] and the fast phase-correlation algorithm (FPC) [12,13]. Many studies exist in the literature concerning the accuracy and precision performance of these algorithms [12–14], although most of these studies concentrate mainly on spectral shifts due to uniform strain loads. External loads, however, can also lead to non-uniform strain field distributions along the sensor grating. When this happens, the original FBG reflected spectrum becomes distorted and shows multiple peaks, as well as more or less pronounced side lobes. In some cases, the spectrum distortion can be used to identify the strain distribution over the sensor [9,15,16], but most often, the distortion is considered as a source of error in the interrogation process. Spectral distortion is crucial for many practical applications. In FBG sensors embedded in composite materials, for example, the amount of spectrum distortion can be quite severe. In this case, the distortion level depends on different factors, such as the size of the FBG and the relative orientation between the FBG sensor and the composite fibers, although most of the spectral distortion arises during the cooling stageof the curing process, when birefringence effects occur because of thermal shrinkage [17–19]. Distortion can also be associated with the presence of structural damage, such as debonding, cracks and delaminations [20–22]. However, spectral distortion is not limited to embedded sensors and has been observed also in surface-mounted sensors. Suaréz *et al*. [23] experienced spectral distortion, while using surface-mounted FBGs to measure transient and residual strains during a welding process. De Pauw *et al*. [24] showed peak broadening and distortion in FBG sensors glued on the surface of a nuclear fuel pin and exposed to conditions similar to those encountered in a heavy liquid metal reactor.

The impacts of FBG spectral deformation on the interrogation performance has been investigated a few times. These studies can be grouped into two main categories: the first focusing on the reconstruction of the non-uniform strain field in static or quasi-static condition [16,25] using neural networks and genetic algorithms; the second dealing with the development of new types of interrogator systems with higher performance in terms of accuracy and interrogation speed [26,27]. However, to the best of our knowledge, no study has been conducted so far to evaluate the performances of different peak detection algorithms when dealing with distorted spectra and dynamic external loads. In 2013, Webb *et al*. [27] performed dynamical strain measurements of embedded FBG sensors using a fast interrogator with full spectrum acquisition. In that case, however, the centroid (CD) was the only algorithm used to process the acquired deformed spectra and to calculate the dynamic average strain.

In this paper, we present a survey on the performance of four peak detection techniques, MD, CD, CC and FPC, when applied to dynamic measurements of distorted FBG spectra. It must be noticed that each of these demodulation algorithms can be selected independently from the type of spectral interrogator used. We compare the performance on the basis of both simulated and experimental data. The transfer-matrix method [28] is used to simulate the behavior of a single-mode FBG sensor subjected to 300 different scenarios of non-uniform strain field distributions along the sensor axis. For each simulation, we evaluate the amount and type of distortion using two indices: the full width at half maximum (FWHM) and the unbalance (UNB). These indices, which are defined in Section 3.2, provide information on the peak broadening, on the intensity reduction and on the asymmetry of the spectrum.

For the experimental analysis, we have mounted three FBG sensors with different FWHM and UNB indices on the surface of a steel plate. In order to introduce distortion, we applied a non-uniform transverse load to the gratings by means of a second smaller plate bolted on top of the sensors. By increasing the tightness of the bolts in 10 consecutive steps, we were able to induce 11 different amounts of spectral deformation. Between two consecutive steps, we performed vibration measurements using a shaker and a full-spectrum interrogation routine, and we compared the SNR levels obtained with the four demodulation algorithms. We conclude on the achievable signal to noise ratio (SNR) and accuracy of the different interrogation algorithms.

The paper is further structured as follows. Section 2 recalls the principles of the detection algorithms compared in our study. Section 3 presents the transfer-matrix method and summarizes the simulation results, while Section 4 deals with the experimental measurements. Finally, Section 5 contains our concluding remarks.

## 2. Demodulation Algorithms

As mentioned in the Introduction, when broadband light encounters a FBG sensor, part of its spectrum at a specific wavelength is reflected. This wavelength is called the Bragg wavelength λ*_B_*, which depends on the effective index *n*_eff_ of the fiber and on the grating period Λ. An external strain field applied to the sensor modifies both *n*_eff_ and Λ and, therefore, affects the reflection spectrum. If this strain field is uniform, then the FBG reflection spectrum only shifts in the amount proportional to the applied strain (assuming isothermal conditions). However, when the strain is non-uniform, the spectrum shifts and distorts [18,21,23,27,29] at the same time. By tracking the changes occurring in the reflection spectrum, the strain distribution can be retrieved. To accomplish this task, many demodulation schemes have been developed. Some of these algorithms simply detect shifts of the Bragg wavelength, while other techniques also take into account the shape of the reflection spectrum. In this paper, we compare the performance of four of these algorithms when they have to deal with distorted spectra and dynamic load measurements. The algorithms that we have selected are the maximum detection (MD) algorithm, the centroid detection (CD) algorithm, the cross-correlation (CC) algorithm and the fast phase-correlation (FPC) technique. The following subsections recall the working principle of each of these algorithms.

### 2.1. Maximum Detection Algorithm

The maximum detection algorithm searches for the wavelength corresponding to the maximum power in the reflection spectrum. It is a pure peak detection algorithm in the sense that it does not take into account the shape of the spectrum. Compared to other methods, the MD algorithm is more sensitive to noise and provides lower levels of accuracy and precision [12,14]. Even if it is easy to implement, it is not the fastest algorithm, especially when used in combination with additional routines that provide sub-wavelength resolution. The MD used in this paper computes the wavelength of maximum reflectivity using the following equation:

(2) λmax=argmaxλ{Rp(λ)}

where λ is the wavelength and *^p^R*(λ) indicates the spectrum obtained with a *p* point quadratic interpolation around the peak wavelength of the original reflection spectrum *R*(λ). Note that the benefit introduced by the sub-interpolation almost vanishes for values of *p* that are too low (not enough interpolation points) or too high (too many interpolation points far from the maximum location and corresponding to lower values of reflected power). In this paper, we assumed *p* = 7 as a good trade-off value.

### 2.2. Centroid Detection Algorithm

The centroid detection algorithm computes the wavelength λ*_c_* corresponding to the geometrical centroid of the reflection spectrum by means of the following equation:

(3) $$\lambda_{c}=\frac{\sum_{j=1}^{Q}\;\lambda_{j}\;R\left(\lambda_{j}\right)}{\sum_{j=1}^{Q}R\left(\lambda_{j}\right)}$$

where *R*(λ*_j_*) is the intensity of the reflection spectrum at the *j*th wavelength position λ*_j_*. The summation can be extended to the entire reflection spectrum or limited to part of the measured spectrum. In the first case, *Q* = *N*, where *N* is the total number of sampling points; in the second case, *Q < N*. This last case (*Q* < *N*) has to be preferred when dealing with almost undistorted spectra with a narrow peak region. Since, in this paper, we focused on spectra with different amounts of distortion, we assumed *Q* = *N*.

### 2.3. Cross-Correlation Algorithm

Another scheme for demodulating of FBG reflection spectra uses the cross-correlation algorithm [11]. In this algorithm, the wavelength shift Δλ between two reflection spectra *R*(λ) and *R*′(λ) = *R*(λ+Δλ) is computed by tracking the peak of the Gaussian distribution obtained by cross-correlating the two spectra. In this paper, the value of Δλ is calculated by implementing the following equation:

(4) $$\Delta \lambda =\underset{\lambda }{\arg\max}\left\{{\text{F}}^{-1}\left[R\left(\lambda \right)*R\prime \left(\lambda \right)\right]\right\}$$

where the symbol * indicates the cross-correlation product and *ℱ*^−1^ is the inverse Fourier transform.

### 2.4. Fast Phase-Correlation Algorithm

The fast phase-correlation has been recently proposed in the literature [12]. Given two spectra *R*(λ) and *R*′(λ) = *R*(λ + Δλ), the FPC computes the shift Δλ by means of the following equation:

(5) $$\Delta \lambda \;=\;\underset{2\leq k\leq M}{\text{median}}\;(\left(∠ℜ\prime \left(k\right)-∠ℜ\left(k\right)\right)\frac{Nk\delta \lambda }{2\pi })$$

where ℛ(*k*) and ℛ′(*k*) are the Fourier transforms of *R*(λ) and *R*′(λ) = *R*(λ + Δλ), respectively, *k* is the generic Fourier spectral line, *M* is the maximum number of Fourier spectral lines considered in the analysis, the symbol ∠ indicates the phase of the complex number and *N* is the number of samples used for each spectrum. The value of *M* can be much lower than *N* without considerably affecting the algorithm performance. In this article, we assumed *M* = 15 as a good trade-off between execution speed and algorithm accuracy and precision.

## 3. Simulations and Performance Analysis

To analyze the performance of the demodulation algorithms introduced in the previous section, we carried out simulations using the commercially available software, MATLAB^®^[30] (The Mathworks, Natick, MA, USA). Subsection 3.1 introduces the transfer-matrix [28] method used to calculate the FBG spectral response as implemented in MATLAB, and Subsection 3.2 illustrates the methodology adopted to process the simulated data and to compare the performance of the different techniques.

### 3.1. Simulation of FBG Distorted Spectra under Steady-State Vibration

**Figure 1.**
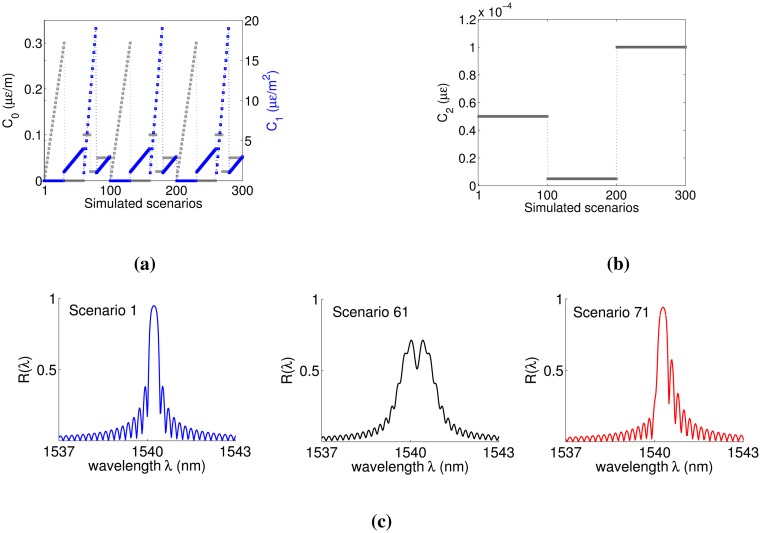
*C_o_, C*_1_ (**a**) and *C*_2_ (**b**) coefficients and distorted spectra (**c**) as a function of the simulated scenarios. (**a**) *C_o_, C*_1_ coefficients. The *C*_0_ coefficient varies from zero to 0.3 *μ*ϵ/*m*, while *C*_1_ goes from zero to 19 *μ*ϵ/*m*^2^. Both *C*_0_ and *C*_1_ repeat identically every 100 scenarios; (**b**) *C*_2_ coefficient. The *C*_2_ coefficient varies from 5 × 10^−5^ *μ*ϵ (Scenarios 1–100) to 5 × 10^−6^ *μ*ϵ (Scenarios 101–200) and 10^−4^ *μ*ϵ (Scenarios 201–300); (**c**) Three distorted spectra scenarios illustrate the different amounts of peak broadening and asymmetry.

To simulate the desired dynamic behavior of distorted FBG spectra, we adopted the transfer-matrix method. In this approach, the grating is divided into short periodic segments, each characterized by a transfer-matrix based on the coupled-mode theory [28]. The characteristics of entire grating are obtained by multiplying the transfer-matrices of all of the short segments. Assuming a grating of length *L* subdivided into *m* periodic segments, the transfer matrix formulation can be computed as:

(6) $$\left[\begin{array}{c} R\left(-L/2\right)\\ S\left(-L/2\right) \end{array}\right]=\prod_{r=1}^{m}T_{r}\times \left[\begin{array}{c} R\left(L/2\right)\\ S\left(L/2\right) \end{array}\right]$$

where *R* and *S* are, respectively, the amplitudes of the reflected and transmitted modes in the FBG axial direction *z*, while *T_r_* is the *r*th transfer matrix. The components of *T_r_* are calculated from the following set of equations [28,29]:

(7) $$T_{r}=\left[\begin{array}{cc} \cosh\left(\alpha \Delta z\right)-i\frac{k_{dc}}{\alpha }\sinh\left(\alpha \Delta z\right) & -i\frac{k_{ac}}{\alpha }\sinh\left(\alpha \Delta z\right)\\ -i\frac{k_{ac}}{\alpha }\sinh\left(\alpha \Delta z\right) & \cosh\left(\alpha \Delta z\right)-i\frac{k_{dc}}{\alpha }\sinh\left(\alpha \Delta z\right) \end{array}\right]$$

(8) $$\alpha =\sqrt{{k_{ac}}^{2}-{k_{dc}}^{2}}$$

(9) $$k_{dc}=2\pi n_{\text{eff}}\left(\frac{1}{\lambda }-\frac{1}{\lambda_{D}}\right)+\frac{2\pi }{\lambda }\bar{\delta n_{\text{eff}\;}}$$

(10) $$k_{ac}=\frac{\pi }{\lambda }v\bar{\delta n_{\text{eff}}}$$

where Δ*z* is the length of each segment, *k_dc_* and *k_ac_* are, respectively, the “dc” and “ac” self-coupling coefficients, *n*_eff_ is the effective index modulation, $\bar{\delta n_{\text{eff}}}$ is the “dc” index change spatially averaged over a grating period Λ_0_, λ*_D_* = 2*n*_eff_Λ_0_ is the design Bragg wavelength and *ν* is the fringe visibility. The relation between the wavelength λ*_D_* and the normal strain distribution ϵ*_zz_* along the *z*-axis is:

(11) $$\lambda_{D}\left(z,t\right)=2n_{\text{eff}}\Lambda_{0}\left[1+a\epsilon_{zz}\left(z,t\right)\right]$$

where $a=1-\frac{1}{2}n_{\text{eff}}[p_{12}-\upsilon (p_{11}-p_{12})]$ is the grating gauge factor [29,31], in which *p*_11_ and *p*_12_ are the components of the fiber-optic strain tensor and *υ* is Poisson's ratio. To account for the dynamic and non-uniform nature of the applied strain, we use the following function:

(12) $$\epsilon_{zz}\left(z,t\right)=C_{0}z+C_{1}z^{2}+C_{2}\sin\left(2\pi f_{o}t\right)$$

where *C*_0_, *C*_1_ and *C*_2_ are constant coefficients, *f_o_* is the frequency and *t* indicates the time. The term *C*_0_ *z* + *C*_1_ *z*^2^ produces the distortion of the FBG spectra, while the term *C*_2_ sin(2π*f_o_t*) introduces sinusoidal spectral shifting. In particular, the linear term *C*_0_ *z* is responsible for spectral broadening, while the quadratic term *C*_1_*z*^2^ induces asymmetric distortion [14]. Such a strain field guarantees that the spectral distortion is constant during the vibration. This assumption holds in many practical situations, as shown in [27]. The case of dynamically changing spectral distortions is not analyzed in this paper. We used the above-described procedure to simulate the behavior of an FBG with *L* = 10^−2^ m, Λ_0_ = 10^−6^ m, *n*_eff_ = 1.452, $\bar{\delta n_{\text{eff}}}=1.131\times 10^{-4}$, *ν* = 1, *p*_11_ = 0.121, *p*_12_ = 0.270, *υ* = 0.17. The design Bragg wavelength of the grating in a strain-free state is 1540.02 nm. The wavelength range λ_*max*_ − λ_*min*_ considered in the analysis was 6 nm, the resolution δλ = 80 pm and the total number of sampling points *N* = 75. Such a parameter selection was made in order to achieve a wavelength resolution almost identical to that of the device that will be used in Section 4 for the experimental analyses. A total of 300 different strain scenarios were simulated by changing the coefficients *C*_0_, *C*_1_ and *C*_2_ in Equation (12), as shown in Figure 1. We simulated a sinusoidal vibration at a frequency *f_o_* = 10 Hz for a time period of 1 s and using a time step of 0.001 s. For every scenario, the MATLAB script calculates the value of λ*_D_* at each time instant using Equation (11) and then refreshes the value of *k_dc_* needed for the computation of the reflectivity according to Equation (6). The amplitude of the sinusoidal shift of the design Bragg wavelength depends exclusively on the *C*_2_ coefficient: the higher *C*_2_, the higher the amplitude of Δλ*_D_*(*t*). In particular, the amplitude of Δλ*_D_*(*t*) achieved with the selected simulation parameters was 70.25 pm for the first 100 scenarios, 7.02 pm for the second 100 scenarios and 140.51 pm for the last 100 simulations.

### 3.2. Processing of Simulation Data and Performance Analysis

**Figure 2.**
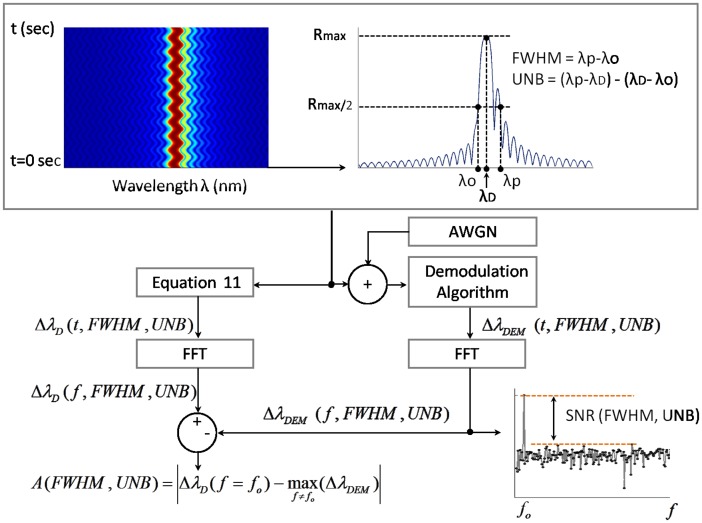
Methodology adopted to process the simulated spectra and to evaluate the performance of the demodulation algorithms.

The procedure adopted to process the spectra corresponding with each simulated scenario is schematically illustrated in Figure 2. Since no additional distortion occurs during the vibration of the spectra, the type of distortion can be preliminary estimated using the following two metrics:

(13) $$\text{FWHM}=\lambda_{p}-\lambda_{o}$$

(14) $$\text{UNB}=\left|\left(\lambda_{p}-\lambda_{D}\right)-\left(\lambda_{D}-\lambda_{o}\right)\right|=\left|\lambda_{p}-2\lambda_{D}-\lambda_{o}\right|$$

where FWHM is the full width at half maximum and UNB is defined here as the unbalance index. λ*_D_* is the design peak wavelength, while λ*_o_* and λ*_p_* are the wavelengths for which the peak power is halved. The FWHM provides information about the spectral width of the peak region, while the UNB measures the amount of spectral asymmetry. Both metrics depend on the selected *C*_0_ and *C*_1_ (Figure 1a). Since *C*_0_ and *C*_1_ repeat identically every 100 scenarios, the FWHM and UNB values repeat in the same way. This means that a total of 100 different couples (FWHM, UNB) were simulated, with the FWHM varying between 0.29 and 3.25 pm and the UNB between 0.0072 and 1.95 pm.

Once the FWHM and UNB indices have been computed, a parallel computational process begins. The first process involves the calculation of the theoretical shift of the design wavelength through Equation (11). The obtained Δλ*_D_* is a function of time and the selected FWHM and UNB. The second process deals with the estimation of the wavelength shifts via the different demodulation algorithm presented in Section 2. First, the simulated spectra are corrupted with white Gaussian noise (AWGN) with a selected SNR value of 40 dB. Then, each demodulation algorithm provides an estimated function Δλ*_DEM_*(*t*, FWHM, UNB), which is successively processed by the FFT algorithm to obtained the wavelength Δλ*_DEM_*(*f*, FWHM, UNB) shift in the frequency domain. To estimate the accuracy of the demodulation algorithm, the amplitude of the computed Δλ*_DEM_*(*f*, FWHM, UNB) is eventually compared with the amplitude of the theoretically-calculated Δλ*_D_*(*f*, FWHM, UNB). At the same time, the dynamic SNR is obtained from the difference between the peak amplitude of Δλ*_DEM_*(*f*, FWHM, UNB) and its noise floor. Accuracy *A*(FWHM, UNB) and SNR(FWHM, UNB) were computed with the following equations:

(15) $$A\left(\text{FWHM},\text{UNB}\right)=\left|\Delta \lambda_{D}{\left(f_{o},\text{FWHM},\text{UNB}\right)}_{dB}-\underset{f}{\max}\left[\Delta \lambda_{DEM}{\left(f,\text{FWHM},\text{UNB}\right)}_{dB}\right]\right|$$

(16) $$\text{SNR}\left(\text{FWHM},\text{UNB}\right)=\Delta \lambda_{DEM}{\left(f_{o},\text{FWHM},\text{UNB}\right)}_{dB}-\underset{f\neq f_{o}}{\max}\left[\Delta \lambda_{DEM}{\left(f,\text{FWHM},\text{UNB}\right)}_{dB}\right]$$

where *f* indicates the frequency, *f_o_* is the frequency of the assumed sinusoidal strain wave (Equation (12)) and the subscript dB indicates the decibels. According to these definitions, low values of *A* combined with high SNR levels indicate better performance. Note that Equation (15) calculates the accuracy *A* as a difference of dB values. From the computed *A* values, the ratio between Δλ*_D_* and Δλ*_DEM_* can be retrieved with the following equation:

(17) $$\frac{\Delta \lambda_{D}}{\Delta \lambda_{DEM}}=10^{\frac{A}{20}}$$

**Figure 3.**
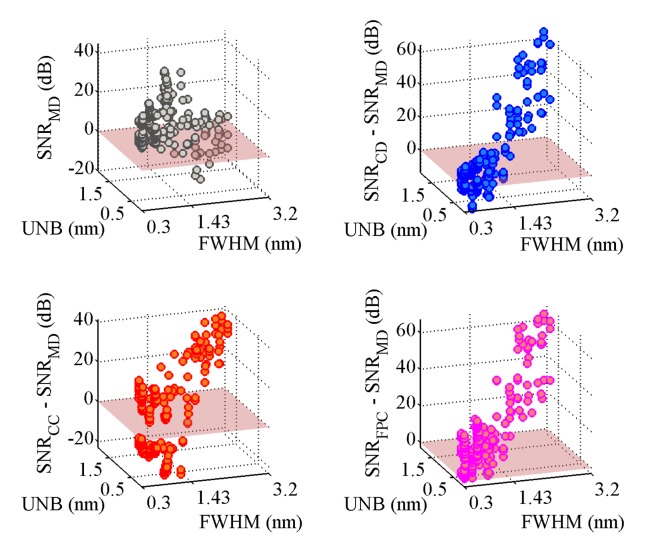
Performance evaluation of the demodulation algorithms in terms of SNR. The maximum detection (MD) algorithm is taken as reference (**upper-right**, **bottom**). For FWHM > 1.43, the probability of failure of the MD algorithm increases. The influence of the asymmetry (unbalance, UNB) on the achievable SNR is limited compared to that of the peak broadening (FWHM).

**Figure 4.**
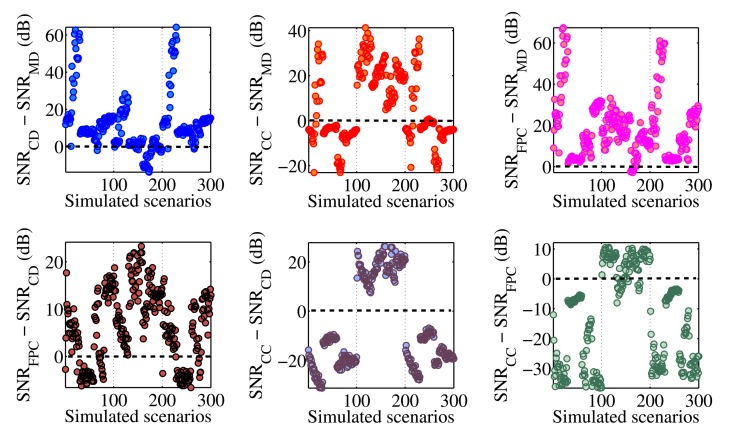
Comparison of the performance based on the variation of the obtained SNRs with the number of simulated scenarios. The SNR levels of CD, CC and FPC are compared with those of the MD algoritm (**upper-row**) and among each other (**bottom-row**). The CC performs better for low-amplitude vibrations (Scenarios 101–200; Δλ*_D_* = 7.02 pm in **upper-center**, **bottom-center**, **bottom-right**). In all other cases, the FPC produces better results than the other algorithms.

Figure 3 shows the obtained SNR levels as a function of the indices FWHM and UNB. The SNR of the MD algorithm is taken as the reference. The first observation is that the SNR of the MD is not always positive, but returns negative values for some couples of (FHWM, UNB) values. From the numerical point of view, this happens because the amplitude of the FFT signal obtained via the MD algorithm lies within the noise floor. In practice, this means that for some simulated scenarios, the MD completely fails in following the dynamical shift of the spectra. In particular, the probability of failing is higher when the FWHM increases beyond 1.43 pm. The other three algorithms always produce positive SNR levels. The CD and FPC techniques allow one to achieve the highest values of SNR. The SNR_CD_ is higher than the SNR_MD_ in 90.3% of the cases. The differences SNR_CC_ − SNR_MD_ and SNR_FPC_ − SNR_MD_ are positive, respectively, for 45.1% and 98.3% of the simulated scenarios. The MD can produce better SNR than CD and CC for FWHM < 1.65 nm and UNB < 0.81 nm. Table 1 reports the maxima and minima SNR values obtained by each demodulation algorithm during the complete set of simulations. The FPC has the highest values of both minimum and maximum SNR. The CD and CC algorithms guarantee almost the same minimum achievable SNR level, which is slightly above 11.5 dB. Table 1 also reports the percentage of cases for which the CD, CC and FPC algorithms produce SNR levels higher than the MD. Figure 4 compares the performance of the different algorithms considering how the SNR changes as a function of the simulated scenario.

We note that:

- CC is the method that performs better in the scenario interval from 101 to 200. In this interval, the *C*_2_ coefficient is minimum (Figure 1b), as well as the amplitude of the design wavelength shift (Δλ*_D_* = 7.02 pm). This suggests that for practical applications involving low-amplitude vibrations, the CC will likely produce signals with better SNR than other techniques.
- The FPC and the CD have SNR levels higher than the CC when the vibration amplitude increases (scenarios from 1 to 100 and from 201 to 300). The FPC can give SNR levels up to 30 dB higher than the CC.
- The difference SNR_FPC_ − SNR_CD_ is always positive for low-amplitude vibrations (Scenario 101–200; Δλ*_D_* = 7.02 pm) and reaches the maximum value of 22.84 dB. When the wavelength shift increases (scenarios from 1 to 100 and from 201 to 300), the FPC continues to give better results than the CD for 65% of the cases (130 scenarios out of 200). The 86% of the cases for which SNR_CD_ is higher than SNR_FPC_ is related to almost undistorted spectra with values of FWHM and UNB lower than 0.25 and 0.09 nm, respectively.

**Figure 5.**
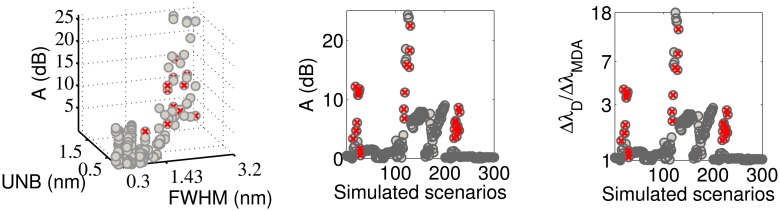
Accuracy of the maximum detection (MD) algorithm as a function of the indices, FWHM and UNB (**left**), and simulated scenarios (**center**). The ratio $\frac{\Delta \lambda_{D}}{\Delta \lambda_{MD}}$ as a function of the simulated scenarios (**right**). The red crosses indicate the scenarios for which the SNR_MD_ < 0.

**Figure 6.**
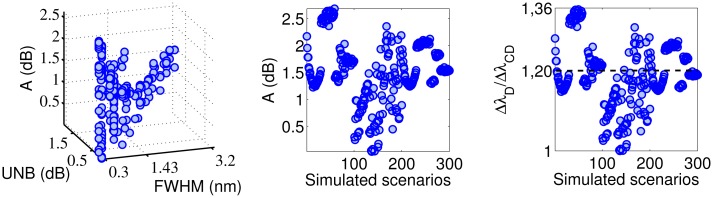
Accuracy of the CD algorithm as a function of the indices FWHM and UNB (**left**) and simulated scenarios (**center**). The ratio $\frac{\Delta \lambda_{D}}{\Delta \lambda_{CD}}$ as a function of the simulated scenarios (**right**).

**Figure 7.**
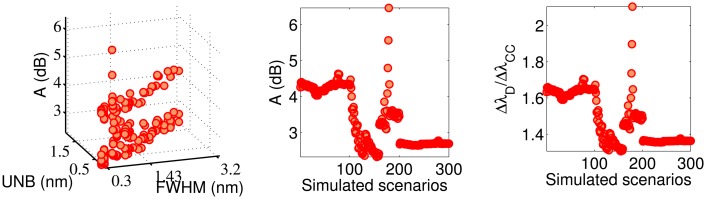
Accuracy of the CC algorithm as a function of the indices FWHM and UNB (**left**) and simulated scenarios (**center**). The ratio $\frac{\Delta \lambda_{D}}{\Delta \lambda_{CC}}$ as a function of the simulated scenarios (**right**).

**Figure 8.**
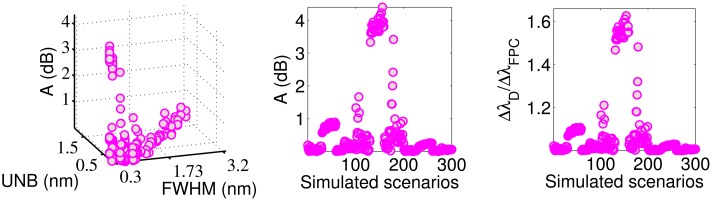
Accuracy of the FPC algorithm as a function of the indices FWHM and UNB (**left**) and simulated scenarios (**center**). The ratio $\frac{\Delta \lambda_{D}}{\Delta \lambda_{FPC}}$ as a function of the simulated scenarios (**right**).

Figures 5, 6, 7–8 show the accuracy of the four algorithms as a function of the FWHM and UNB indices and the simulated scenarios. Figure 5 refers to the MD accuracy performance. The red crosses indicate scenarios for which the SNR_MD_ of Figure 3 is negative. We note that the MD accuracy decreases considerably when the FWHM and the UNB increase. In particular, the worst accuracy occurs at FWHM = 2.48 nm and UNB = 1.63 nm and *C*_2_ = 5 × 10^−6^*μ*ϵ. Although this case corresponds to a positive SNR_MD_, the ratio $\frac{\Delta \lambda_{D}}{\Delta \lambda_{MD}}=18$ indicates that the MD demodulation of the wavelength shift fails. In fact, rather than detecting a sinusoidal Δλ*_D_*(*t*) of amplitude 7.02 pm, it provides an amplitude of 0.39 pm. Statistically, the major number of failures in terms of both SNR and accuracy occurs for FWHM and UNB, respectively, above 1.43 and 0.87 nm.

The accuracy of the CD technique (Figure 6) shows low variation with both FWHM and UNB. In 59% of the simulated scenarios, the CD accuracy error is lower than 20 % ( $\frac{\Delta \lambda_{D}}{\Delta \lambda_{CD}}<1.2$) . If we consider low-amplitude vibrations (Scenarios 101–200), the percentage increases from 59% to 85%. The CD is always more accurate than the CC (Figures 6 and 7), while it is less accurate than the FPC (Figures 7 and 8) in 260 of the 300 simulated scenarios (86.67%). Particularly, for *C*_2_ > 5 × 10^−6^*μϵ* (Scenarios 1–101 and 201–300), the FPC accuracy is always better (*i.e.*, lower) than the CD. The ratio $\frac{\Delta \lambda_{D}}{\Delta \lambda_{FPC}}$ is lower than 1.2 for 89% of the cases and lower than 1.05 for 72% of the cases. The effect of FHWM and UNB on the FPC accuracy is limited.

## 4. Experimental Results and Discussion

**Figure 9.**
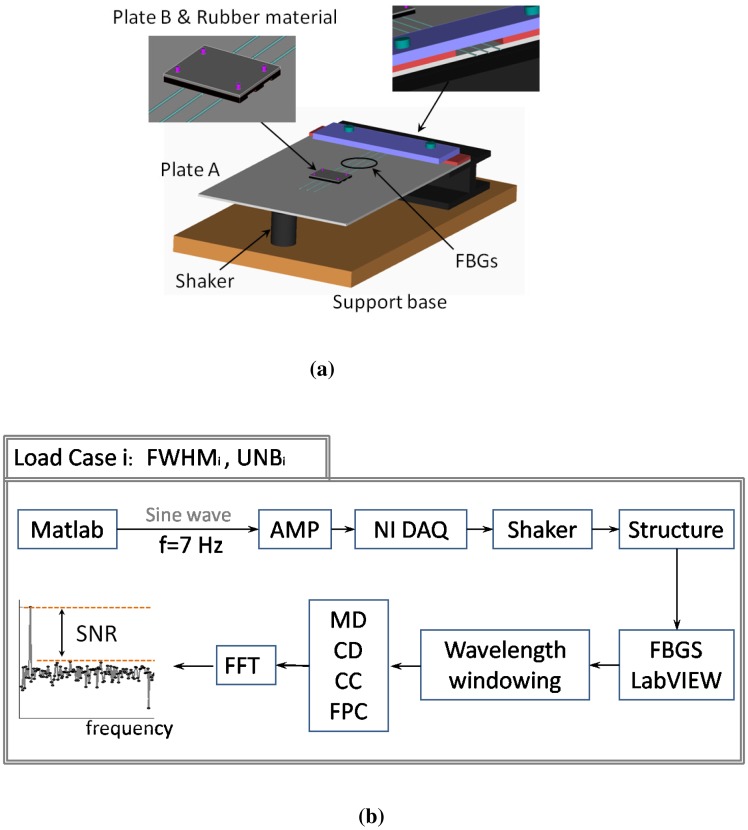
Experimental setup (**a**) and procedure (**b**) used to compare the performance of the different algorithms in terms of the achieved SNR. (**a**) Experimental setup; (**b**) Schematic diagram of the experimental procedure.

**Figure 10.**
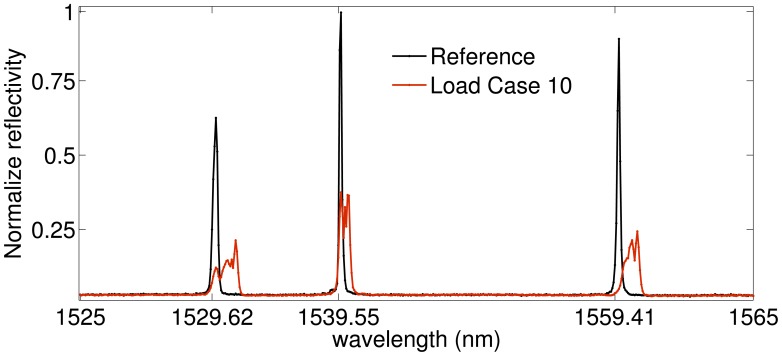
Normalized reflectivity of fiber Bragg grating 1 (FBG1) (1529.62 nm), FBG2 (1539.55 nm) and FBG3 (1559.41 nm) for two different transverse load conditions. Spectral distortion occurs when the load increases from the reference condition (black curve) to Load Case 10 (red curve).

**Figure 11.**
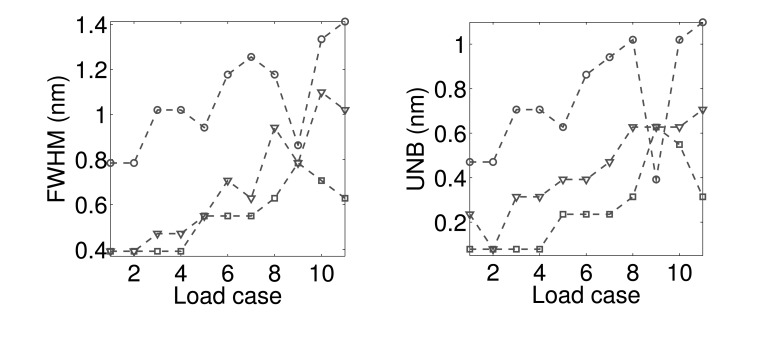
FWHM **(left)** and UNB **(right)** indices *vs.* load case for FBG1 (circle), FBG2 (square) and FBG3 (triangle). The maximum FWHM obtained is 1.41 nm.

**Figure 12.**
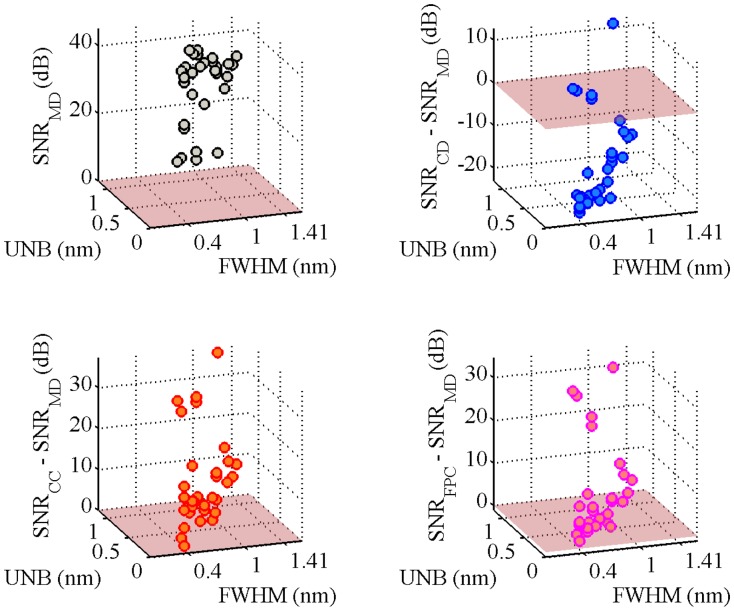
Performance evaluation of the demodulation algorithms based on experimental SNR levels. The maximum detection (MD) algorithm is taken as reference **(upper-right, bottom).** The FPC **(bottom-right)** performs better than the other algorithms.

**Figure 13.**
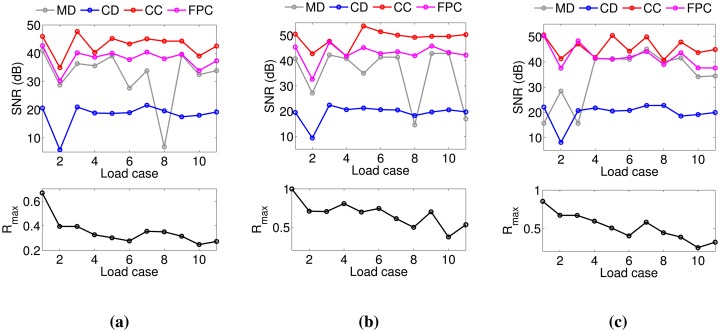
SNR levels **(top)** and maximum reflectivities **(bottom)** variations as a function of the load case. (**a**) FBG1; (**b**) FBG2; (**c**) FBG3.

To validate the simulated results obtained in Section 3, we carried out a performance analysis on the basis of experimental data. To compare the performance of the four demodulation algorithms for several experimental conditions corresponding to different FWHM and UNB values, we designed with the setup described in Figure 9a. Three FBGs (FBG1, FBG2, FBG3) with Bragg wavelengths 1529.62 nm (FBG1), 1539.55 nm (FBG2) and 1559.41 nm (FBG3) are used. They have grating length L = 10^−2^ m and initial FWHM of 0.7843 nm (FBG1), 0.3923 nm (FBG2) and 0.3921 nm (FBG3). The FBGs are glued to the surface of a steel plate (Plate A in Figure 9a) with dimensions of 21 cm × 19 cm × 0.1 cm. The plate is clamped along one of its shorter edges and attached to a shaker at the opposite edge. In order to produce distorted spectra with several FWHM and UNB indices, a second smaller plate (Plate B in Figure 9a) of dimensions of 5 cm × 3 cm × 0.1 cm is mounted on top of the sensors and fixed to the main plate with bolts (magenta cylinders in Figure 9a). A rubber material is included between the gratings and Plate A. This material has two tasks: it protects the fibers, and at the same time, it induces a distortion of the spectrum. When the bolts are tightened, the tooth profile of the rubber material section (Figure 9a, upper left) applies a non-uniform transverse load to the gratings. As a consequence, the original FBG spectra distort: the original peak regions become broader, and multiple peaks arise due to birefringence effects [32,33]. Figure 9b explains the experimental procedure used to analyze the algorithm performance. First, a reference transverse load is selected by arbitrarily choosing an initial level of bolt tightness. Then, a sinusoidal wave of amplitude 4 V and frequency 7 Hz is generated in MATLAB, amplified and sent to the the shaker through an NI USB-6341 data acquisition card [34]. The vibrating spectra of each FBG sensor are multiplexed in one broader spectrum and recorded using a commercially available FBGS FBG scan 700 [35] interrogator (wavelength range 1525–1565 nm and resolution 78 pm) in combination with an in-house developed LabVIEW [36] code. Three wavelength windows of a bandwidth of 7 nm are then applied to the recorded data. The first window is centered around the original Bragg wavelength of FBG1 (1529.62 nm); the second and the third windows are centered, respectively, around 1539.55 nm (λ*_B_* of FBG2) and 1559.41 nm (λ*_B_* of FBG3). In this way, the spectral vibration associated with each FBG is retrieved and processed using the MD, CD, CC and FPC algorithms. The SNR levels are eventually computed from the FFT of the calculated wavelength shifts Δλ. Once the SNR values are stored, the tightness of the bolt is manually increased, and a new transverse load case corresponding to spectra with different FWHM and UNB is generated. The vibration measurements are then repeated and the new SNR levels computed. In this paper, we analyzed 11 different load cases (including the reference load condition). Figure 10 shows a comparison between the original (Load Case 1 = the reference) spectrum obtained by multiplexing the three FBG reflected spectra and the equivalent spectrum corresponding to Load Case 10. The variation of the FWHM and UNB indices of each sensor as a function of the load case is reported in Figure 11. It is worth noticing that, in this procedure, no sensor was adopted to measure the applied transverse load. At the same time, the load was manually increased since uniform load distributions were not required. Highly non-uniform load conditions worked better for our purposes, because they induced more spectral distortion. Figure 12 shows the experimental SNR levels obtained by the different demodulation algorithms. The MD is taken as the reference. Figure 13 shows for each of the three FBG sensors the evolution of the SNR levels obtained with the four demodulation algorithms together with the evolution of maximum reflectivity. Overall, the results are in good agreement with the simulated SNR levels in Figure 3.

In particular, we note that:

- The MD algorithm produces SNR levels that are always positive. This is due to the fact that the maximum FWHM (1.41 nm) obtained from the experiments is below the critical value 1.43 nm, above which, according to simulations, the MD algorithm starts to fail.
- The SNR of the CD is generally worse than the SNR of the MD. This is in accordance with the simulations (Figure 3; Scenarios 101–200 in Figure 4) and is due to the limited values of FWHM, UNB and wavelength shift (Δλ_max_ = 4.75 pm) achieved in experiments.
- CC and FPC perform better than MD and CD. The levels of SNR_CC_ − SNR_MD_ and SNR_FPC_−SNR_MD_ in Figure 12 are very similar to those reported in Figure 3 for FWHM < 1.41 nm and UNB < 1.09 nm.
- The CC algorithm performs better than the FPC, with SNR values from 1.4 to 9.8 dB higher. This is in accordance with what we saw in our simulations, where the CC worked better than the FPC for spectral vibrations with an amplitude below 7.02 pm (Scenarios 101–200 in Figure 4).
- The variability of the SNR levels as a function of the reduced maximum reflectivity is higher for the MD algorithm rather than for CD, CC and FPC. The CC and the FPC produce SNR levels that are always higher than 30 dB, independent of the maximum reflectivity values.

It is worth noticing that a one-by-one comparison between simulation and measurements is not possible for several reasons. First of all, the simulated strain functions do not match the applied strain used for the measurements exactly. In fact, in the simulation, a longitudinal strain function is assumed, while in the measurements, a transverse load is applied. Therefore, in the experiments, the birefringence effect is much more pronounced than in the simulation and affects with a different weight the SNR levels. Secondly, the amplitude of the vibration achieved during the experiments does not exactly coincide with either of the three simulated amplitude levels (*C*_2_ coefficients in Figure 1b). The amplitude of the excitation plays an important role in terms of the SNR performances of the different analyzed algorithms. The peak locking effect [12], for instance, depends on the vibration amplitude (*i.e.*, wavelength shift) once the wavelength resolution is fixed. Therefore, different wavelength shifts are associated with different amount of performance degradation due to the peak locking effect. Moreover, the noise levels used to corrupt the simulated FBG spectra are not selected to exactly represent the noise incorporated in the measurements. In addition to what was already stated, it has to be mentioned that the experimental set-up used in this paper was not conceived of to apply controllable loads in a repeatable manner. It was rather conceived of in order to allow a fast and easy application of non-uniform loads. Of course, as it is, the set-up allows one to repeat the measurements, although not with the same applied transversal load. Statistically speaking, however, the experimental results are able to confirm the trends identified by the simulations. In fact, even if there are additional influences on the achievable SNR levels, because of birefringence, measurement noise and vibration amplitude, the performance of the algorithms as a function of the spanned FWHM and UNB ranges follow the same trend showed by the simulations. For a one-by-one comparison, a more sophisticated set-up could be prepared. At the same time, different strain functions capable of better simulating the birefringence effect could be developed. Moreover, additional distortion indices sensible to both asymmetry and the amount of birefringence could be used.

## 5. Conclusions

In this paper, we presented a comparison of four demodulation algorithms for dynamical measurements of distorted FBG spectra. These algorithms were the maximum detection (MD), the centroid detection (CD), the cross-correlation (CC) and fast phase-correlation (FPC). Using the transfer-matrix method, we first simulated the dynamical behavior of spectra with different levels of distortion. To classify the amount and type of distortion, we used the full width at half maximum (FWHM) and the unbalance (UNB) indices. These two indices provided information about the broadness of the peak region and about the amount of spectral asymmetry, respectively. The performance of the algorithms was evaluated in terms of accuracy and SNR. We also carried out experimental measurements to validate our simulations. Our results show that:

- The maximum detection algorithm is the most sensitive to distortion. More particularly, the probability that this algorithm fails at retrieving the actual wavelength shifts increases for values of FWHM > 1.43nm.
- The fast phase-correlation algorithm yields the best combination of high SNR and accuracy.
- The fast phase-correlation and centroid detection algorithms are the most accurate, but the fast phase-correlation produces higher SNR levels and works better when the amount of spectral distortion increases and the vibration amplitude decreases (*i.e.*, when Δλ is low).
- The cross-correlation technique has the highest performance in terms of SNR for low-amplitude vibrations (*i.e.*, low wavelength shifts Δλ).
- The symmetric distortion (FWHM) associated with peak broadening and intensity reduction affects the performance of the algorithms more than the asymmetric distortion (UNB).

Note that the performance analysis presented in this paper assumes that a number of model parameters remain fixed, such as the wavelength bandwidth, the number of points used for interpolation in Equation (2) and the values of Q and M in Equations (3)–(5). A different selection of these parameters could lead to changes in the performance. In particular, according to our experience, the MD is very sensitive to both the wavelength bandwidth and the number of points used for sub-wavelength interpolation. The CD algorithm is also sensitive to wavelength bandwidth, although less than the MD. In addition, the CD performance can considerably change when a different number of points Q is used to compute the centroid of the reflection spectrum. On the other hand, the changes in the FPC performance due to a different selection of M (the number of Fourier spectral lines) are moderate. Moreover, the FPC, as well as the CC algorithms have low sensitivity to wavelength bandwidth. These considerations demonstrate that correlation-based algorithms are more versatile than conventional techniques, although not necessarily more accurate. This paper has shown that the FPC is the algorithm that guarantees the best combination of versatility, precision and accuracy.

## Figures and Tables

**Table 1.** Maxima and minima SNR levels obtained by each demodulation technique for the 300 simulated scenarios (Rows 1 and 2). The centroid detection (CD) and the fast phase correlation (FPC) yield a higher SNR for more than 90% of the simulated scenarios, while the cross-correlation (CC) performs better than maxium detection (MD) only for 45.1% of the cases.

	**MD**	**CD**	**CC**	**FPC**
SNR_max_ (dB)	44.59	51.80	42.75	56.33
SNR_min_ (dB)	−16.48	11.63	12.42	18.92
$\frac{\#({\text{SNR}}_{\text{DEM}}-{\text{SNR}}_{\text{MD}}>0)}{300}(\%)$	0	90.3	45.1	98.3
